# The need for community-controlled tools to monitor health impacts of housing and living conditions in Australia

**DOI:** 10.3389/fpubh.2025.1509550

**Published:** 2025-03-26

**Authors:** Rosemary Wyber, Kate Summer, Ingrid Stacey, Samantha Stiles, Judith Katzenellenbogen, Asha C. Bowen, Rachel Burgess

**Affiliations:** ^1^Wesfarmers Centre of Vaccines and Infectious Diseases, The Kids Research Institute Australia, University of Western Australia, Nedlands, WA, Australia; ^2^Yardhura Walani, Australian National University, Acton, ACT, Australia; ^3^Cardiovascular Epidemiology Research Centre, The University of Western Australia, Nedlands, WA, Australia; ^4^Cardiology Population Health Laboratory, Victor Chang Cardiac Research Institute, Darlinghurst, NSW, Australia; ^5^Department of Infectious Diseases, Perth Children’s Hospital, Nedlands, WA, Australia; ^6^ Wonnarua

**Keywords:** housing policy, Healthy Living Practices, Aboriginal and Torres Strait Islander people, community health, program evaluation, data sovereignty

## Abstract

Despite millennia of strong and continuous culture, inadequate housing has profound consequences on the health and wellbeing of Aboriginal and Torres Strait Islander people in Australia. For example, the excessive and inequitable burden of childhood skin infections, rheumatic fever, gastrointestinal disease and ear infections can all be linked to failures in housing policy, funding and maintenance. Aboriginal and Torres Strait Islander communities and peak bodies continue to call for greater community control and investment in housing. A commonality among stakeholders in this otherwise complex and contested space is the association between poor housing and poor health, and the need to evaluate the health impacts of housing improvement initiatives which speak literally to this connection, e.g., *Housing for Health* [New South Wales (NSW), 1997-current], *Fixing Houses for Better Health* (National, 2005–2009), and *Healthy Homes* [Northern Territory (NT), 2021]. We explore the contemporary landscape of housing investments and initiatives seeking to improve health outcomes among Aboriginal and Torres Strait Islander people in Australia, as well as the dearth of quality evidence and agreed approaches to evaluation. We outline the need to develop a monitoring tool grounded in routinely collected primary care data which will provide community-controlled organizations with sovereign capacity to measure health outcomes associated with housing. This would in turn inform political accountability and scale-up of Indigenous housing initiatives that work.

## Housing and health in Australia

The fundamental relationships between housing, health and wellbeing are well-established, and high standards in all domains have been achieved for most people in Australia (1, 2). However, colonization, racism and socioeconomic marginalization continue to drive particularly inadequate housing, in terms of both quality and quantity, for Aboriginal and Torres Strait Islander people (3–6). This is especially true in remote areas where housing stock is low, access to maintenance services is reduced, and existing dwellings are subject to chronic disrepair (3, 7–9). Aboriginal and Torres Strait Islander people are six times more likely to rely on social housing than non-Indigenous people (10, 11) and many families have little choice but to live in culturally and climatically unsuitable, overcrowded^1^ homes with under-or non-functioning health hardware, damp and mold, extremes of temperature, and safety issues (9, 12). Despite good awareness, these conditions preclude people from undertaking healthy behaviors, or Healthy Living Practices (HLPs), with profound impacts on health and wellbeing (3, 13, 14).

In 1987, the landmark Uwankara Palyanku Kanyintjaku (UPK) Report defined nine HLPs that substantiate the specific elements of the home/living environment needed to enable Aboriginal and Torres Strait Islander people to live healthy lives (13). The HLPs, including the capacity to wash people and remove wastewater safely, have strong pathways to the prevention of infectious diseases, in addition to non-communicable diseases and wellbeing more broadly (14, 15). The harmful impacts of inadequate HLP capacity for individuals and communities span human rights, cultural, economic, social, wellbeing and physical health outcomes, and have thus spurred a range of investments and initiatives by communities, the private sector, and all levels of government in Australia. However, the burden of implementing the HLPs and the impact of failing to do so is placed largely on Aboriginal and Torres Strait Islander communities.

## Investments and initiatives to improve housing and health for Aboriginal and Torres Strait Islander people

Leadership by The Coalition of Aboriginal and Torres Strait Islander Peak Organizations (Coalition of Peaks) and the National Aboriginal and Torres Strait Islander Housing Association has spearheaded renewed Federal and State/Territory Government-level commitments toward improvement in housing supply, maintenance and management for Indigenous people (16). Delivered under a multitude of different agreements across all States and Territories, total national investment in Aboriginal and Torres Strait Islander housing over the next 10 years is expected to exceed $7 billion (17–25). This includes the 2024 announcement of a joint $4 billion investment from the Federal and NT Governments to build new houses in remote NT under the program *Our Community, Our Future, Our Homes* (23, 25). Broad-reaching investments to address general shortages in social and affordable housing have also been made in most jurisdictions (26–29). In addition to categorical investment in housing, various environmental health/infrastructure development programs supporting HLPs for Aboriginal and Torres Strait Islander communities are ongoing (30–35). Notwithstanding the promise of ostensive investments, the policy environment is ever-changing and difficult to navigate and mechanisms for ongoing accountability and evaluation within and between policy/funding cycles are lacking.

Recent announcements to improve Aboriginal and Torres Strait Islander housing have been companioned by new partnerships and commitments to the co-management of housing with community-controlled organizations, including Aboriginal Housing NT, Aboriginal Land Councils, Aboriginal Building Enterprises, and Indigenous Land and Sea Corporations (23, 24). The central role of these organizations is critical to increasing Indigenous voice and authority within what is otherwise Australia’s paternalistic and ideological model of complex, and ultimately failing, Indigenous housing reform (4). Where the strength and sovereignty of Aboriginal and Torres Strait Islander people are recognized, there is cause for celebration, including the Jalbi Jiya (‘Your Home’) program in Broome (36), and other home ownership pathway programs (37), Wilya Janta (‘Standing Strong Together’) housing collaboration in Tennant Creek (38), and the Pemulwuy Project for affordable housing in Sydney (39). Ability to measure what is working well is critical to scale up of these successes and inform the co-design of others.

## The unmet need to evaluate the health impacts of housing and living conditions

Different approaches to improve housing are likely to have different effects on health and not all strategies will be equally efficient or effective (40). Tools to understand these differences in outcomes and to inform decision-makers are under-developed. Proxy measures of inputs (such as the amount of money spent) or outputs (such as the number of new houses constructed or maintenance works completed) are common but blunt reporting tools (40, 41). They present a simplified and fleeting view of housing policies, especially in remote areas where high construction costs often result in under-specification (42),^2^ and do not directly correlate with health indicators. The absence of agreed program logic and measurement approaches stymies monitoring and evaluation and contributes to the systemic gaps in understanding the short, medium and long-term health impacts of changes to housing and living conditions. The inability to readily quantify the true effects of housing on health perpetuates a fragmented approach to decision-making that is not evidence based, and hinders innovation and scale-up of effective initiatives. Lack of community-controlled measures also perpetuates reliance on organizational research and excludes many Aboriginal and Torres Strait Islander people from the entire process of data collection, communication and decision making.

Embedded within housing investments and initiatives are hopes and claims that they will improve health. For example, the Federal Government’s Fixing Houses for Better Health (FHBH) program was able to make key HLP–related improvements to over 2000 houses in 34 communities between 2005 and 2009 at a cost of $5.5 billion (40). However, the program did not cater for the collection of data linking housing improvements to health indicators, such that an audit found that it was “not possible to draw relationships between the implementation of the FHBH program activities and its overall purpose of improving Indigenous health” [(40), pp. 20]. Similarly, as part of the review of the Federal Indigenous Community Housing and Infrastructure Program (CHIP), which was subsequently abolished upon recommendation of the review, stakeholders identified that accountability was related to outputs as opposed to outcomes and that processes were followed but resultant improvements in health were unclear (43); it was “easier to spend the money than to set objectives and monitor outcomes” [(43), pp. 14]. Performance indicators for the National Partnership Agreement on Remote Indigenous Housing and the Remote Housing Strategy ($5.4 billion, 2008–2018) were focused on numbers of dwellings, occupancy rates, incidence of homelessness, completion of repair and maintenance works, and municipal service provision, but not health and wellbeing (44, 45). More recently, an evaluation of a large Healthy Homes Program in the NT demonstrated improvements in home function relative to HLPs but was unable to include any quantitative data on associated health outcomes (46). The July 2024 Closing the Gap annual report concluded that housing for Aboriginal and Torres Strait Islander people is not on track to meet targets, and that data was not available to establish or measure access to and quality of housing and essential services (47).

There are increasingly urgent calls for improved monitoring capacity among primary care providers and for monitoring tools to be held by community. In Queensland, the *Our Place First Nations Housing and Homelessness Action Plan* states that “First Nations-led monitoring and evaluation are essential elements in supporting shared accountability for outcomes, identifying opportunities for improvement, learning and adaptation, and driving effective investment” [(20), pp. 22] but acknowledges that the necessary frameworks and tools are yet to be developed. Additionally, the Queensland Government’s *Aboriginal and Torres Strait Islander Environmental Health Plan*, calls for improved collaboration between primary healthcare providers and the environmental health workforce to address infectious diseases, and states that “More work is required to ensure consistent and measurable gains in environmental health outcomes including the use of sound program intelligence and reliable performance indicators” [(48), pp. 6]. The 2022 review of the Western Australian Aboriginal Environmental Health Program included recommendations to develop and apply quality outcome indicators and a robust reporting framework, including “appropriate monitoring of outcomes and outputs using service, primary health care and hospital data” [(49), pp. 5]. A recent article by Stoneham et al. (50) recommends the development of a list of mutually agreed conditions using hospital data to assist environmental health clinical referral processes.

Where attempts to evaluate the health impacts of housing improvement programs *have* been made, there is a reliance on hospitalization data. Although hospitalization data can be cost-effective and suitable in the absence of other tools/approaches, it has limitations in the housing context. For example, following learnings from the FHBH program, the landmark *Housing for Health* program in NSW was able to demonstrate a significant reduction in hospital separations for four infections (respiratory, skin, gastrointestinal, and ear) following repair and maintenance works made to Aboriginal community housing between 1998 and 2009 (51, 52). This is arguably the most important case study in the Australian context to date demonstrating that a standardized housing testing and repair methodology can have sustainable associated health benefits that are measurable by way of hospitalization data (52). However, the infectious disease outcomes used in *Housing for Health*, and in fact most other housing/HLP-related conditions, are diagnosed and managed mostly by local community clinics (53–58). By contrast, hospitals are usually located in larger urban centers and hospitalization data are more severe, less common indicators capturing serious illness and under-reporting these primary care concerns [e.g., see (59, 60)]. Consequently, hospital-based metrics are likely to significantly underestimate the true effect of housing/HLP improvements at a community scale. Supplementing hospitalization data with routinely collected primary care data could paint a more complete picture and affirm the critical role of Aboriginal and Torres Strait Islander Community Controlled Health Organizations (ACCHOs) in managing diseases related to housing and living conditions.

Academic studies have incorporated a range of different methods to measure the impact of housing/HLP-capacity on the health of Aboriginal and Torres Strait Islander people. A systematic review of infectious diseases associated with inadequate housing and Healthy Living Practice capacity in Australia companions this article (Prospero registration CRD42024541393). Most studies use a mix of self-reported and researcher-collected data, for example, the NHMRC-funded Housing Improvement and Child Health study explored the effects of housing construction programs in 10 remote NT communities between 2003 and 2004 by asking primary carers of children aged 0–7 years to self-report on experiences of five childhood illnesses which were related to the functional state of infrastructure gleaned from physical assessments of households (61). Whilst academic studies have helped to understand the impacts of inadequate housing/HLP-capacity on health, similar designs are not suitable for prospective longitudinal monitoring or routine program evaluation because they rely on intensive data collection within a research framework. Also, differences in research designs and their reproducibility mean that data are not directly comparable, spatially or temporally. The consistency and sustainability of evaluation and monitoring is of utmost importance, since improving both housing and health for Aboriginal and Torres Strait Islander people is a long-term, intergenerational commitment.

## Foundational data sovereignty and self-determination

Individuals and communities having genuine self-determination and control is a key intermediary between housing and health. Alongside sound evidence, good policy and adequate funding, is a concomitant priority for community organizations, namely ACCHOs and Aboriginal community housing providers, to partner in evaluating the impact of housing initiatives. This reflects priority reforms in Closing the Gap, particularly *Priority Reform Two: Building the Community Controlled Sector* and *Priority Reform Four: Shared Access to Data and Information at a Regional Level* (62). It also reflects the National ACCHO *(NACCHO) Policy Position Paper: Aboriginal Housing for Aboriginal Health* (2021) (4) which identified the need to “Implement a rigorous national research, evaluation and data collection program that monitors the impact of Aboriginal and Torres Strait Islander housing policy against health indicators” as one of 12 key priorities, in addition to the transfer of management to Aboriginal community-controlled organizations. Further, the *Maim nayri Wingara Indigenous Data Sovereignty Principles* make clear the need for data which is “relevant and empowers sustainable self-determination and effective self-governance” [(63), pp. 2]. Operationalizing these priorities means moving beyond research that investigates the relationships between housing and health to focus on the development of a prospective approach to data collection which is designed, sourced, interpreted and communicated by and for community organizations to influence robust, integrated policy and investments. The development of a metric indicator in the form of a primary care software tool (hereafter, a monitoring tool) and transition to use should be informed by the best available evidence and input from technical experts but be led, implemented and tested by Aboriginal and Torres Strait Islander people and organizations as the primary end users.

## The development of a tool to monitor housing-associated disease is proposed

A monitoring tool that measures health outcomes responsive to housing/HLP-capacity could improve service delivery, ensure accountability of new government partnerships to Aboriginal and Torres Strait Islander organizations, and support community-led programs. We recommend that this tool be:

- Grounded in routinely collected primary care data capturing presentations of an agreed bundle of conditions associated with inadequate housing/HLP-capacity,
- Replicable in methodology over time and place, and validated with well-defined applications and limitations, and
- Generated, interpreted, held and communicated by community organizations.

A comprehensive monitoring tool to evaluate the health effects of housing reform would ideally span communicable disease, non-communicable disease and social and emotional wellbeing (SEWB). However, more work is needed before associations between housing and non-communicable disease or SEWB can be considered definitive. Conversely, associations between infectious disease and housing are relatively well-understood and could be developed into a meaningful tool for near term use. The feasibility of this approach is supported by similar indicative conditions and evaluation tools used to monitor performance and price funding of public hospitals in Australia [e.g., hospital-acquired complications (64) and avoidable hospital readmissions (65)]. In-depth primary care data coding/analysis and clinical validation studies are a focus of future work and publications.

## Limitations

One of the reasons for the failure to evaluate the health impacts of housing initiatives is the complexity of the relationship. A focus on infectious diseases provides a practical delineation, although multi-factorial influences on health (e.g., sociodemographic and cultural factors, service access, and service-seeking imperatives), and impacts on SEWB would remain uncaptured by the proposed tool until a time that it could be expanded to include them. This risks further entrenching a reductionist, biomedical, lens on Aboriginal and Torres Strait Islander housing issues.

Finally, Aboriginal and Torres Strait Islander housing and environmental health are contested spaces (3). Data are powerful and political, particularly for marginalized communities who are underrepresented in data collection and decision-making (66). Simultaneously, clinical/statistical data are often overvalued relative to lived experience and cultural knowledge (67). Development of a monitoring tool potentially offers a new level of transparency for housing/HLP-improvement programs which variably intersects with stakeholder priorities. Invariably, it requires an acknowledgment of these realities and a commitment to reflective research, in service to community priorities. Consensus-based decision-making processes will help to mitigate some challenges and potentially elucidate others and will nonetheless require thoughtful engagement with a wide range of stakeholders and end-users. We hope that prospectively describing the rationale for our ongoing work and the values underpinning the process will support trust, constructive dialogue and collaboration.

## Conclusion

Housing and HLP capacity for many Aboriginal and Torres Strait Islander people remains inadequate, with significant impacts on health and wellbeing. There are increasing commitments to address this, alongside a resounding call among all stakeholders for improved monitoring of health outcomes. Identifying indicative infectious diseases could be a first step to developing a monitoring tool that is grounded in primary care and led by communities.

## Data Availability

The original contributions presented in the study are included in the article/supplementary material, further inquiries can be directed to the corresponding author.

## Footnotes

^1^Reasons for household crowding are structural as well as cultural. The negative impacts of crowding (e.g., wear and tear on health hardware; stress and disease) can co-exist with the positive aspects of being together.

^2^It should be noted here, that high standards of housing *are* achievable in remote areas with adequate resourcing and strategic planning, as demonstrated by the mining sector.
